# Chloral Sleep in the Treatment of Chorea
1Med. Rec., April 1.


**Published:** 1899-05-06

**Authors:** 


### CHLORAL SLEEP IN THE TREATMENT OF CHOREA.[1]

Dr. Lichtschein, of New York, has for some years employed chloral hydrate, in doses sufficient to produce a somnolent condition, in the treatment of neurasthenia in conjunction with the Weir-Mitchell rest cure, and he has noticed that during such treatment appetite increases, digestion and assimilation improve, and, as there is no other kind of energy expended, a rapid gain in weight and strength, and a remarkable abatement of nervousness and irritability, result. Taking into consideration then the fact that choreic movements generally cease during sleep, and that for some time after a night's rest they are weaker, he decided to extend the use of chloral sleep to the treatment of chorea. In the small number of cases in which he employed this method the treatment was successful, no evil after-effects were noticed, while during the continuance of the rest by chloral the patients gained on an average nearly half a pound a day. Of course this use of chloral is not original, and, among other cases, reference may be made to a remarkable one recorded by Sir W. T. Gairdner, in which while treating a girl, aged eight years, with 20 grains of chloral three times a day, a treble dose was accidentally given, with the result that the patient was unduly drowsy for 24 hours, but all the spasm absolutely disappeared, and, what was more curious, this freedom from spasm continued even after every trace of the action of the drug had disappeared, and there was no return of the chorea.

In one of Dr. Lichtschein's cases a boy, nine years old, suffering from chronic chorea, was given fifteen grains of chloral hydrate with gr. $\frac{1}{200}$ of sulphate of strychnine every two to three hours. He was still somewhat restless, so the chloral was increased to 20 grains. This latter dose caused a deep sleep, the boy grew quiet, and the twitching ceased. On the third day, the sleep being very deep, the chloral was reduced to gr. 15, and then to gr. 10, every two or three hours. For the first two days there was no desire to eat, although when food was given it was taken mechanically; but on the third day the boy grew hungry, and after that he ate greedily during his prolonged sleep. On the tenth day he weighed three pounds more than on the first. From the fifteenth day he received diminishing doses of chloral until the eighteenth day, when the drug was stopped. On that day he was eight pounds heavier than at the beginning of the treatment; the choreic movements had ceased entirely, and they did not return.

[1] Med. Rec., April 1.

